# Measuring Online Wellbeing: A Scoping Review of Subjective Wellbeing Measures

**DOI:** 10.3389/fpsyg.2021.616637

**Published:** 2021-03-11

**Authors:** Zhen Xin Ong, Liz Dowthwaite, Elvira Perez Vallejos, Mat Rawsthorne, Yunfei Long

**Affiliations:** ^1^School of Medicine, University of Nottingham, Nottingham, United Kingdom; ^2^Horizon Digital Economy Research Institute, University of Nottingham, Nottingham, United Kingdom; ^3^National Institute of Health Research (NIHR) Biomedical Research Centre for Mental Health and Technology, School of Medicine, University of Nottingham, Nottingham, United Kingdom; ^4^Institute of Mental Health, University of Nottingham, Nottingham, United Kingdom; ^5^School of Computer Science, University of Essex, Colchester, United Kingdom

**Keywords:** subjective wellbeing, online wellbeing, wellbeing measures, systematic review, positive affect, negative affect, life satisfaction

## Abstract

With the increasing importance of the internet to our everyday lives, questions are rightly being asked about how its' use affects our wellbeing. It is important to be able to effectively measure the effects of the online context, as it allows us to assess the impact of specific online contexts on wellbeing that may not apply to offline wellbeing. This paper describes a scoping review of English language, peer-reviewed articles published in MEDLINE, EMBASE, and PsychInfo between 1st January 2015 and 31st December 2019 to identify what measures are used to assess subjective wellbeing and in particular to identify any measures used in the online context. Two hundred forty studies were identified; 160 studies were removed by abstract screening, and 17 studies were removed by full-text screening, leaving 63 included studies. Fifty-six subjective wellbeing scales were identified with 18 excluded and 38 included for further analysis. Only one study was identified researching online wellbeing, and no specific online wellbeing scale was found. Therefore, common features of the existing scales, such as the number and type of questions, are compared to offer recommendations for building an online wellbeing scale. Such a scale is recommended to be between 3 and 20 questions, using mainly 5-point Likert or Likert-like scales to measure at least positive and negative affect, and ideally life satisfaction, and to use mainly subjective evaluation. Further research is needed to establish how these findings for the offline world effectively translate into an online measure of wellbeing.

## Introduction

A single, standardized definition of wellbeing does not currently exist in the literature. Previous attempts to define wellbeing could be argued to be a “description,” rather than a “definition,” as they focus on the dimensions of wellbeing rather than wellbeing itself (Dodge et al., 2012). Nevertheless, it is important to consider how wellbeing is conceptualized in research. There are several different perspectives on what wellbeing is and how to test it. In psychological research, Alexandrova (2015) identifies three schools of thought, each with different underlying philosophical theories. The first refers to a “hedonic balance” — the balance of positive and negative emotions in someone. The second is life satisfaction, which is related to a subjective judgement of one's own life: people care about a range of things (work, basic needs, comfort, activism, etc.), and the feelings from those things make up life satisfaction. The third perspective refers to a slightly altered version of Aristotle's idea of eudaimonia. It includes a sense of autonomy, mastery, purpose, and connectedness to people, as well as “flourishing” as a person (Ryff, 1989; Alexandrova, 2015). In the field of positive psychology, the two main traditions of study are the Hedonistic and Eudemonic traditions (Deci and Ryan, 2008). This review focuses on subjective wellbeing (SWB), which comes from the hedonic tradition and includes life satisfaction. The term subjective is used because it allows for individuals to define what brings them pleasure and what is important to their happiness (Alexandrova, 2005)—it is wellbeing according to what the person says it is. The definition of SWB used in this paper includes three components: positive affect, negative affect, and life satisfaction (Diener and Suh, 1997).

Life satisfaction (LS) is a cognitive-judgmental process (Diener et al., 1985), in which a person judges their own life according to their own criteria for an ideal life (Shin and Johnson, 1978). It is important to highlight that a person's “ideal life” is a standard set by the individual themselves and not a standard imposed externally (Diener et al., 1985). Positive and negative affect refer to the positive and negative emotions and moods that a person feels (Diener and Suh, 1997). Someone experiencing positive affect may describe their experience with words such as “joy,” “pride,” and “affection,” whereas negative affect may be described as “anxiety,” “anger,” or “sadness” (Cacioppo and Berntson, 1999). While their names imply that they are opposites, this is not the case, and they should be treated as two independent dimensions (Cacioppo and Berntson, 1999; Lee and Oguzoglu, 2007). It is possible for someone to be experiencing both positive and negative affect at the same time. A state of good SWB is a person of high positive affect, low positive affect, and high life satisfaction (Deci and Ryan, 2008).

Organizations and governments are increasingly interested in measuring wellbeing; for example, The World Happiness Report is an annual report by the United Nations Sustainable Development Solutions Network (SDSN) that reviews evidence on the science of happiness and wellbeing and ranks countries by how “happy” they are (Helliwell et al., 2020). Furthermore, The World Health Organization (WHO) has published the WHOQOL-100 (Skevington, 1999) and WHOQOL-BREF (WHO, 1996) as measures of quality of life and wellbeing. For public policy, there are three main purposes of measuring wellbeing: monitoring progress, designing policy, and appraising policy (Dolan et al., 2011). Measuring wellbeing helps monitor a nation's “happiness” and the efficacy of policy decisions that are meant to improve the wellbeing of a nation. Relying on measures of wealth such as national income may be inaccurate for approximating the wellbeing of a nation (Layard, 2010). Thus, the importance of measuring wellbeing is unquestionable. How wellbeing is measured and what should be measured, however, are heavily contested issues (Diener et al., 1999, 2018; Ryan and Deci, 2001; Alexandrova, 2005; Deci and Ryan, 2008). Since 1999, over 170,000 articles on the topic of wellbeing have been published (Diener et al., 2018). For researchers, the topic of evaluating wellbeing presents numerous scientific, philosophical, and intellectual challenges that, if solved, could have widespread socioeconomic impacts and give further insight into some of the mysteries of why we do what we do.

There are numerous scales and measuring tools for wellbeing. While generic scales exist, for example, the WHO-5 Wellbeing Index (WHO-5) (Topp et al., 2015), which asks respondents to rate statements such as “*Over the last two weeks I have felt cheerful and in good spirits*,” there are a wide range of contextual scales that take particular circumstances into account. For example, the PostTrans Questionnaire, an SWB measure for postnatal mothers with type 1 diabetes mellitus, includes statements such as “*I am coping well with looking after both my baby and my diabetes*” (Rasmussen et al., 2013); the Teacher Subjective Wellbeing Questionnaire (TSWQ), a SWB measure for teachers, includes statements such as “*I feel like people at this school care about me*” (Renshaw et al., 2015). As such, an online wellbeing scale (OWS) would be a scale developed for the online context, measuring the effects of the online world on a person's wellbeing. It does not mean simply a measure of wellbeing that is delivered via online means (online questionnaires, etc.) but might include questions such as “*How would you rate the social support you receive from social media?*” It is important to consider why an OWS specifically for the online world is needed.

The UK Office for National Statistics (ONS) reports that 99% of 16–44 year olds in the UK were recent internet users at the time of survey, and only 7.5% of adults in the UK have never used the internet in 2019 (Office for National Statistics, 2019). With such a large proportion of people on the internet, it is important to assess the effects of the online world on wellbeing. It cannot be simply assumed that the results from SWB research offline translates directly to online contexts. Take social support as an example. Social support is one aspect that influences SWB (DeNeve, 2016) and is frequently measured by SWB scales. A Canadian survey of 5,000 adolescents noted that while the number of offline friends are positively correlated with SWB, the number of online friends has no correlation to SWB (Helliwell and Huang, 2013). On the other hand, a report by the Pew Research Centre noted that US Facebook users had slightly more close relationships and reported getting more social support than the US average (Hampton et al., 2011), although they did not investigate wellbeing directly. This suggests that interactions on an online social network do not mimic the interactions of an offline social network, and so the applicability of offline SWB scales that include questions on social support is questionable.

A systematic narrative review (Best et al., 2014) found a variety of results with regards to how the online world may influence wellbeing. Notably, evidence was found that supports the notion that the online world helps adolescents connect and develop social support networks; however, there was also evidence that online communication practices may have a negative relationship with wellbeing. It also highlights a growing number of studies that suggest the relationship between internet usage and SWB is complicated. Another study found that administering an educational program to teach internet use to 22 elderly participants in Israel resulted in participants feeling happier about their current quality of life, less depressed, less lonely, and a greater sense of empowerment (Shapira et al., 2007). Although not enough evidence to make any concrete conclusions, the study does suggest that, among older adults, it is possible that simply engaging with computers or the internet can result in changes to a person's wellbeing.

Following traditional patterns of measuring wellbeing may also lead to a skeuomorph. A skeuomorph is “an ornamental version of something that was, in an earlier product, a functional necessity. Fake shutter sounds in digital cameras” (Pogue, 2013). It is something from the past kept around because of familiarity. Wellbeing measures based purely on factors from a conventional wellbeing scale would limit the potential scope and applications (Schueller et al., 2013); it may include irrelevant factors transferred from the offline world and miss important nuances of the online world. Trying to apply an SWB scale from offline to online, while not necessarily pointless, could be ineffective.

The objective of this paper is to answer the research question: *What does measuring subjective wellbeing entail, and how is this applied to the online context?* It describes a scoping review of studies that use a scale to measure SWB, with the aim of identifying the pertinent features of such scales and how they are applied and particularly how they are used to study online wellbeing.

## Methods

### Design

A scoping review was undertaken, using the methodological framework of Arksey and O'Malley (2005), to identify the extent, range, and nature of research on a topic and identify gaps in the literature. The population identified was studies involving adults aged 18 years and over, and comparators are the number of items in the scale, the types of questions asked, how they are scored, which SWB components were measured, the period of time measured, and whether measures are entirely subjective or alternative measures were used. The outcomes are the wellbeing scales, and the study was designed to identify all studies using a scale to measure subjective wellbeing, independently of its quality. Preferred Reporting Items for Systematic Reviews and Meta-Analyses (PRISMA) reporting guidelines for scoping reviews were followed (Moher et al., 2009).

### Data Sources

The search for the literature was limited to English-language peer-reviewed articles published between 1st January 2015 and 31st December 2019. The electronic databases MEDLINE, EMBASE, and PsychINFO were searched for studies using SWB scales or measurement instruments.

### Search Strategy

A brief, informal search for existing systematic reviews on wellbeing measurement instruments was carried out on Google Scholar using the search “*well?being measuring instruments systematic review.”* One systematic review was identified from this search (Lindert et al., 2015). The search terms were reviewed, along with those in another review already known to the authors (Schrank et al., 2013). Following this, a brainstorming session took place between all the authors to identify the search strategy used in this paper, which is as follows:

[(index or measure^*^ or scale? or clinimetric? or metric? or questionnaire? or survey? or interview? or assessment? or inventor^*^or tool? Or indicator^*^or indices or subscale^*^) and well?being].ti.

Titles only were searched because it was felt that it was more likely that this would focus the search on studies that were specifically looking at scales. Words describing the type of wellbeing (i.e., “subjective”) were not included as some studies refer to SWB as simply “wellbeing,” leading to potentially missed studies. The term “happiness” was also excluded, as it is often a vague term with multiple meanings (Diener et al., 2018).

### Data Extraction

The web application Rayyan QCRI was used to screen articles by title and abstract (Ouzzani et al., 2016). Article records were screened by two researchers independently, with any disagreement being resolved by discussion. Articles were then screened by full text and all included articles used in the qualitative analysis. Critical appraisal of study quality was not performed and is not expected of scoping reviews (Pham et al., 2014). All studies were searched for scales used to measure wellbeing. Questions on the scale were read and assessed for inclusion or exclusion by one researcher. Table 1 summarizes the basic inclusion and exclusion criteria used: SWB scales were included, and scales for other types of wellbeing including eudemonic wellbeing were excluded. Scales including both eudemonic wellbeing and SWB were included; however, questions from the scales judged to be eudemonic in nature were removed from further analysis. Scales measuring depression or anxiety symptoms were also excluded, unless studies that included those scales were clearly using them to measure negative affect; negative affect refers to mood and emotions (Diener and Suh, 1997), while depression/anxiety symptoms include symptoms such as fatigue/low energy, poor concentration, poor or increased appetite, etc. (WHO, 2015).

**Table 1:** Inclusion and exclusion criteria for scoping review.

**Inclusion criteria**	**Exclusion criteria**
All study types	Gray literature
English-language studies published in a peer-reviewed journal between 01/01/2015 and 31/12/2019	Unpublished articles
Studies using SWB scales, including validation, development, and assessment	Studies using scales intended for clinical diagnoses of psychiatric illnesses, e.g., depressive disorder, bipolar disorder
Studies using SWB as an outcome measure	Studies that make no mention of SWB or SWB measuring instruments
	Studies not specific to humans
	Studies looking at types of wellbeing other than SWB, including but not limited to sexual, economic, philosophical, eudemonic.

### Data Analysis

All included scales were then analyzed for the comparators listed in *Design*. Common themes between scales were coded by one researcher, and the frequency of those themes were recorded. A subgroup analysis was also planned for studies that included scales or research related to online wellbeing. Descriptions of what scales were intended to measure, as stated by paper authors, were taken into consideration but were not strictly adhered to during analysis. This is due to a lack of agreement in the literature as to what certain terms actually mean, and a lack of a systematic way of defining those terms (Huta and Waterman, 2014); thus, one scale calling their measures “Quality of life” may mean “Life satisfaction” in another scale, causing unpredictability and variability. Instead, one researcher judged whether scales should be included, and what they measured, based on rigorous definitions agreed between all authors. This decision was taken to improve consistency of definitions. Definitions of the terms “SWB,” “Positive affect,” “Negative affect,” and “Life satisfaction” are described in *Introduction*.

## Results

### Study Selection

**Figure 1:**
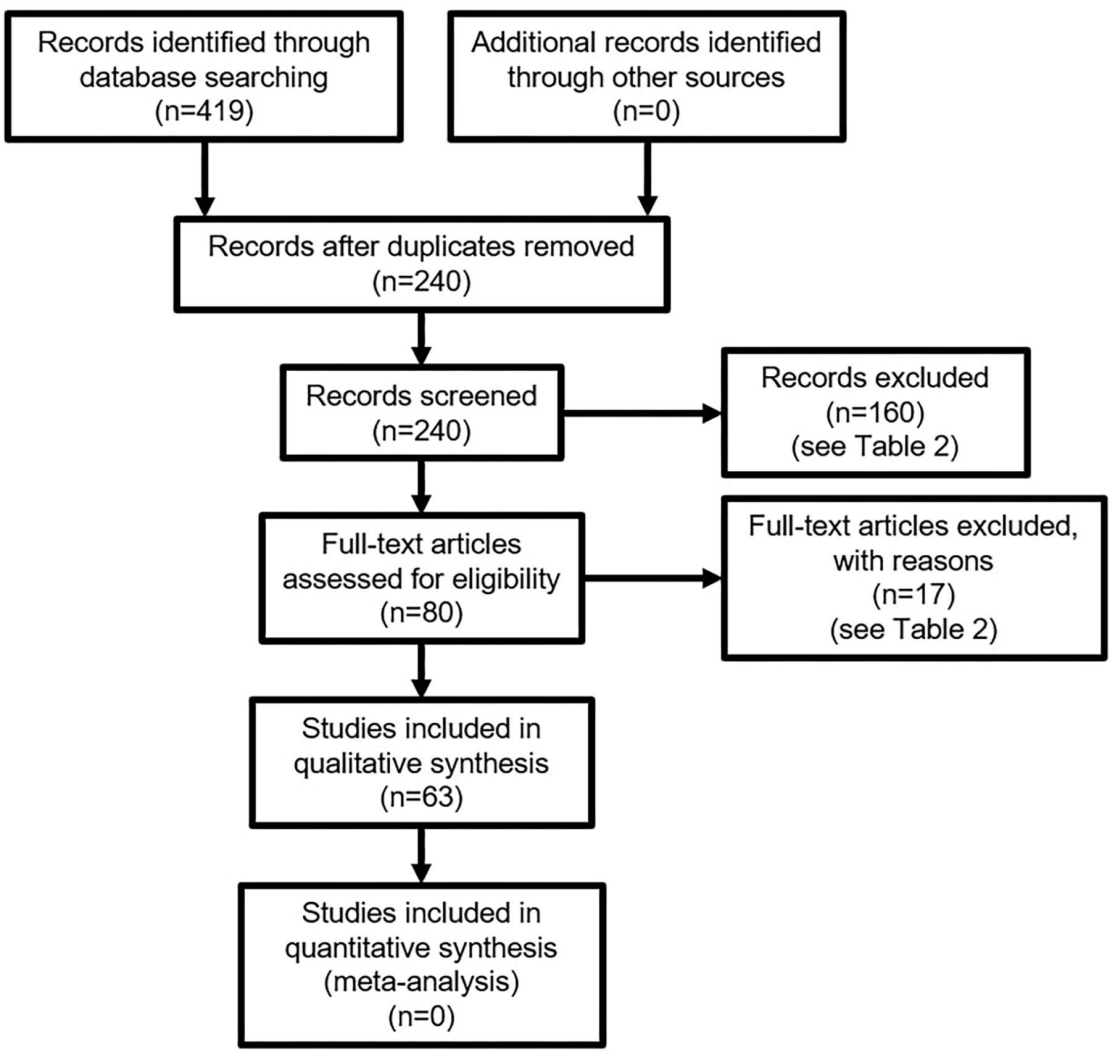
PRISMA Flow Diagram.

A total of 63 full-text articles were included in the search for scales (see Figure 1); reasons for exclusion are summarized in Table 2. From these articles, 56 scales were identified, of which 38 scales were included, and 18 scales excluded, in the analysis. A full list of included and excluded scales is provided in the Supplementary Material.

**Table 2:** Summary of excluded studies.

**Reason for exclusion**	**Record excluded**	**Full text excluded**
Not general SWB (e.g., wellbeing in children or workplace, sexual or spiritual wellbeing)	74	4
Conference abstracts	26	
No mention of measuring SWB or using SWB measurement tools	20	2
Clinical or mental health measures not for the purpose of investigating negative affect (depression, anxiety, etc.)	20	6
Eudemonic wellbeing measures only	12	4
Wellbeing in animals	4	0
Book chapters	2	0
News articles or opinion pieces	1	0
Correction article	1	0
Full text not available	0	1

The most used scales were the Warwick–Edinburgh Mental Wellbeing Scale (WEMWBS) (Tennant et al., 2007), which was used in 12 studies; the Personal Wellbeing Index (PWI) (Cummins et al., 2003), used in 10 studies; and the Positive Affect Negative Affect Schedule (PANAS) (Watson et al., 1988), General Health Questionnaire-12 (GHQ-12) (Goldberg and Williams, 1988), and WHO-5 (Topp et al., 2015), which were all used in 5 studies. The remaining scales were used in between one and four studies. The top five most used scales all had papers published regarding their development. Of all the 38 included scales, 33 have peer-reviewed journal papers published on their development, 4 have had guidance published but no peer-reviewed journal papers on their development were found, 1 of the scales was published only in a book with no references in any journal paper, and 3 scales had no information published on their development.

The main sources of bias are publication bias (gray literature was excluded), studies were not appraised for quality, and one researcher performed the analysis. These are discussed in greater detail in *Limitations*.

### Synthesized Findings

The following sections report common features of all the included scales, including how scales are structured, what components of SWB they measure, and how they measure it. Percentages on some tables may not add up to 100% because some scales can be counted under multiple categories. See Additional Material for (1) full list of included and excluded scales and (2) validation and evaluation status of included scales.

### How Subjective Wellbeing Scales Are Structured

The mean number of questions was 13 (median 10), with a minimum of 1 and a maximum of 37. Most of the 38 scales (57.9%) had up to 10 questions, and only 18.4% had more than 20 questions. The vast majority of scales used Likert or Likert-like measures (92.1%), with that being the only type of question used for most (86.8%); one also used interviews, and one used Likert, Yes/No, and multiple-choice questions. Only one measure used only multiple-choice questions and one only Yes/No questions, while one had variable question types depending on researchers choices, using the Experience Sampling Method (ESM) (Larson and Csikszentmihalyi, 2014). Of the 35 scales that used Likert-scales, 2 used more than one, to give a total of 40 measures; the American Time Use Survey (Stone et al., 2018) uses both 6- and 10-point Likert scales, and the European Social Survey (Huppert et al., 2009) uses 4-, 5-, 6-, 7-, and 11-point scales. The mean number of points on the Likert scales was 6 (median, 5), with a minimum of 3 and maximum of 11. Most measures used 5-point Likert scales (30.0%) or 4-point Likert scales (25.0%).

Scales also differed on whether they used positive or negative scoring (see Table 3). Positive scoring means that a high score indicates good wellbeing, while negative scoring means that a high score indicates poor wellbeing. Most scales used positive scoring (39.5%), although a few use negative scoring (21.1%). Some scales (7.9%) do not use a scoring system, for example giving a code instead and providing categorical data for analysis; for example, the EuroQoL−5 Dimensions−3 Levels (EQ-5D-3L) survey gives codes such as “112233” (EuroQol Group, 1990). Additionally, some scales (21.1%) used reverse scoring, with some questions scored in the opposite direction to the other questions, also shown in Table 3. Reverse scoring was applied for questions that were in a nature opposite to the scoring. For example, of the positive scoring scales, 66.7% (26.3% of all scales) did not use reverse scoring, but 26.7% (10.5% of all scales) applied it to negatively worded items. For the scales that used reverse scoring, it was used for positive affect items in half of them and negative affect items in half of them.

**Table 3:** Scoring systems used by scales, *N* = 38.

		**Reverse scoring (%)**						
		**No**	**For positive items**	**For negative items**	**Unknown (scoring system not found)**	**N/A**	**Varies throughout scale**	**Total (%)**
High Score Indicates	Good wellbeing (positive scoring)	26.3	–	10.5	2.6	–	–	39.5
	Poor wellbeing (negative scoring)	5.3	10.5	–	2.6	2.6	–	21.1
	No scoring	–	–	–	–	7.9	–	7.9
	Varies by subscale	5.3	–	–	2.6	–	–	7.9
	Different scoring systems exist	5.3	–	–	–	–	–	5.3
	Varies throughout scale	–	–	–	–	–	2.6	2.6
	Unknown (scoring system not found)	5.3	–	–	10.5	–	–	15.8
	**Total (%)**	47.5	10.5	10.5	18.3	10.5	2.6	

### Which Subjective Wellbeing Components Are Measured

Table 4 summarizes the components of SWB that each scale measured. Positive Affect and Negative Affect were both included in 50.0% of scales, although they only appear together in 28.9%. The third component of SWB, Life Satisfaction, was included in just 15.8% of scales; all three components commonly accepted to make up SWB only appeared together in 5.3%. Depression or anxiety symptoms were included as a proxy intended to measure negative affect in 18.4% of scales. As such, negative measures were most common. One scale had a variable set of components, as above.

**Table 4:** Components of subjective wellbeing measured, *N* = 38.

**Components measured**	**% of Scales**
Negative affect only	21.1
Positive affect only	18.4
Life Satisfaction only	10.5
Positive affect and negative affect	23.7
Positive affect, negative affect, and life satisfaction	5.3
Positive affect, life satisfaction, and depression/anxiety symptoms	2.6
Depression/anxiety symptoms only	15.8
At researcher's discretion	2.6

### How Subjective Wellbeing Components Are Measured

The scales varied on whether they asked about a specific time period or for a global evaluation of a respondent's life. For example, the Subjective Happiness Scale asks respondents for a global evaluation, asking them to rate themselves on a 7-point Likert-like scale for the statements such as “In general, I consider myself,” with 1 being “not a very happy person” and 7 being “a very happy person” (Lyubomirsky and Lepper, 1999). On the other hand, the UK ONS Wellbeing Scale asks questions for a specific time period, such as “Overall, how happy did you feel yesterday?” (Office for National Statistics, 2018). Table 5 summarizes the time periods covered by the scales. The majority of scales (65.8%) used specified time periods, with 21 scales (55%) only using such scales. The rest relied on a global evaluation, apart from the one scale that used variable scales as above. Of those scales that asked for evaluations within a specific time period, they varied widely in the periods used. These are summarized in Table 6. Five scales (13.2%) asked for more than one time period. For example, the ONS Well-Being questionnaire (Office for National Statistics, 2018) asked for both “in general” and “yesterday,” and the Cantril Self-anchoring scale (Cantril, 1966) asked for “In general,” “Five years ago,” and “Five years in the future,” giving 28 measures of specific time periods. Half of the 28 measures that specified a time did so for the recent past, being the last 2 weeks or more recently, while an additional 14.3% asked for up to the month previously. Three measures (10.7%) referred to future wellbeing. Several measures were quite vague about their time period, for example, “the past few weeks” or “in the near future.”

**Table 5:** Time periods covered by the scales, *N* = 28.

**Time scale**	**% of Measures**
5 years ago	3.6
Past year	3.6
Past 30 days	7.2
Last month	3.6
Past few weeks	3.6
Past 2 weeks	21.4
Past week	17.9
Past 3 days	3.6
Yesterday^*^	3.6
Past day^*^	3.6
Today	3.6
Instantaneous	1.7
Future	3.6
In the near future	3.6
5 years future	3.6
At researcher's discretion	3.6

* *Note that “Past day” and “Yesterday” do not measure the same thing. “Past Day” refers to the last 24 h, whereas “Yesterday” does not include the current day*.

**Table 6:** Global evaluation vs. specific time scales, *N* = 38.

**Time scale**	**% of Scales**
Global evaluation	31.5
Specified time periods	55.3
Both global and specified	10.5
At researcher's discretion	2.6

Most scales used subjective evaluation measures, asking respondents to make a personal evaluation of SWB, such as “*How happy are you?*” (84.2%); 55.3% used only these measures in their scale (Table 7). Some scales used alternative measures (42.1%). Of these scales, daily function, loneliness, sleep quality, and social support were used by 18.8% each (three scales), the ability to deal with problems was measured in 12.5% (two scales), and 6.3% (one scale) each measured alertness, emotional expression, enough energy, enough money, laughter frequency, stressful life events, and time for things they enjoy. Only 13.2% of scales relied solely upon these alternative measures, these being the scales that measures loneliness, laughter frequency, and stressful life events.

**Table 7:** Evaluation methods used by the scales.

**Evaluation method**	**% of Scales**
Subjective evaluation only	55.3
Alternative measures only	13.2
Both subjective evaluation and alternative measures	29.0
At researcher's discretion	2.6

### Online Subgroup Analysis

No scales specifically measuring online wellbeing were found from the scoping review. One study investigated the effects of internet access on various outcome measures including wellbeing (Kearns and Whitley, 2019) and used the WEMWBS, which was also the scale used most overall. To summarize, this scale (Tennant et al., 2007) has 14 questions on a 5-point Likert scale, with a high score indicating good wellbeing and utilizing reverse scoring for negative items only. The scale measures positive affect only, for a specified time period of the past 2 weeks. The scale includes alternative measures of the ability to deal with problems and social support as well as subjective evaluation.

## Discussion

### Measuring Online Wellbeing

The most glaring issue highlighted by the scoping review is the lack of OWS or online wellbeing research published in English articles within the past 5 years. Only one study investigated SWB in an online context (Kearns and Whitley, 2019); it used the WEMWBS (Tennant et al., 2007) in its original form to measure the effects of internet access on various outcome measures including wellbeing. The lack of a standardized, widely accepted, validated online wellbeing scale (OWS) presents a critical gap in the literature. A lack of research into online wellbeing is problematic not least because of the vast majority of adults using the internet frequently. By developing an OWS, researchers can measure online wellbeing in a standardized fashion. This opens the door to research such as the relationship between online wellbeing and overall SWB. A standardized scale means that internal and external validity can be scrutinized, instead of relying on researchers to develop their own metrics, which may or may not be verified. Comparison of such a scale in different online contexts could also help assess which online activities lead to positive or negative effects on wellbeing. Recommendations for policy and public bodies such as the NHS could come as a result. Without a standardized OWS, issues can arise such as problems in comparing results between studies or studies missing out on the finer details of online wellbeing when conventional SWB scales are used instead.

The creation of a validated outcome measure to build studies around in the form on OWS could encourage such research. Studies are currently forced to use existing SWB measures that may or may not be validated for the online world. The WEMWBS for example includes questions on social relationships (“I've been feeling loved” and “I've been feeling close to others”) (Tennant et al., 2007), but online communication may be experienced in a different way or hold different importance for wellbeing online, and there is evidence for both positive and negative effects of online interactions. There exists the Online Social Experiences Measure (OSEM), but this mainly measures social support online (Kent de Grey et al., 2019). Although social support is important when thinking about SWB (Diener et al., 2018), and consequently online wellbeing, it is not the only dimension that should be measured. Therefore, the relation between online and offline social relationships and their effect on SWB remains to be tested (Diener et al., 2018).

Additionally, the “online world” needs to be better operationalized and contextualized, as it could include any application or activity that is connected to the internet including reading the news, social media, multiplayer video gaming, etc. As new immersive technologies are introduced (e.g., virtual reality, augmented reality), this will push the boundaries of what “the online world” is. Moreover, with the arrival of 5G mobile technology, it is expected to further increase consumer demand for online content and services (Ofcom, 2017; Ericsson, 2019). This situation is problematic, and governments and regulatory bodies are increasingly concerned about the harms (e.g., internet addiction, cyberbullying, fake news, radicalization), which people face online every day (DCMS, 2019) and left with no research tools available to systematically assess the impact on wellbeing. For example, the evidence about screen time and wellbeing is inconclusive (Przybylski and Weinstein, 2017), highlighting the lack of standardized measures and methodologies that would support the development of robust study designs. The lack of validated measures leaves researchers without tools to assess the impact that new legislation may have on internet usage and its impact on wellbeing. The UK's Age Appropriate Design Code (i.e., a set of policies to minimize the capture of personal data and maximize privacy by default features among online services accessed by children under 13 years of age) set to become law in the next few months, is one example that illustrates the needs for such tools in order to inform policy makers and regulatory bodies of the impact that new legislation may have on online wellbeing and the reduction of online harms (Information Commisioner's Office, 2020). These are important questions for the future of online and offline wellbeing research.

If questions are not adapted or reworded to fit the online context, it is not guaranteed that online wellbeing is measured. Rewording questions [for example, from “I have been feeling interested in other people” (Tennant et al., 2007) to “I have been feeling interested in other people on social media”] could change the way respondents interpret the question, and so the validity or reliability of the well-established WEMWBS may not be the same. A new OWS is necessary because research is using SWB scales that may not be fit for the purpose. The remainder of this paper discusses findings from assessment of existing SWB measures and recommendations on how these should be applied to the creation of on OWS.

### Scale Structure

Current subjective wellbeing scales have a wide range of lengths: from 1 to 37 questions. For context, there are also scales measuring other wellbeing factors with far more questions, such as the World Health Organization Quality of Life-100 (WHOQOL-100) (Skevington, 1999). The results show 81% of scales had 20 or less questions. This may be because having too many questions increases the risk of respondent dropout or respondents not fully completing the questionnaire. In support of this, shorter postal questionnaire lengths (less questions) have been shown to make response more likely (Edwards et al., 2002). For online questionnaires, it has been found that longer questionnaires had statistically significantly more “don't know” responses and semicompleted questionnaires, suggesting reduced engagement and response quality with longer questionnaires (Deutskens et al., 2004). Shortened scales are often published after originally longer questionnaires, for example, the K-6 (Kessler et al., 2003) after the K-10 (Kessler et al., 2002), the WHOQOL-BREF (WHO, 1996) after the WHOQOL-100 (Skevington, 1999), and SWEMWBS (Haver et al., 2015) after the WEMWBS (Tennant et al., 2007). Thus, questionnaires with too many questions should be avoided or brief versions of questionnaires also developed.

On the other hand, a scale with too few questions risks not measuring “enough” to accurately assess the key components of a person's wellbeing. For example, the Global Life Satisfaction question included as part of the PWI (Cummins et al., 2003) is the only question assessing life satisfaction; it also leaves out positive and negative affect and thus arguably does not sufficiently measure SWB. In summary, a scale that is too long may risk participant fatigue, and a scale that is too short may risk not asking enough information. For these reasons, it is recommended that an OWS be between 3 and 20 questions to maximize its measurement reliability and validity. It should be noted, however, that no research has been found that specifically addresses the topic of ideal length of questionnaire for wellbeing scales, so this may be an avenue for future research.

Likert and Likert-like scale questions were by far the most common, and most measures used only such scales. Why other question types (Yes/No, Multiple choice, etc.) are underrepresented is unclear, but it may be speculated that Likert-type scale questions offer the ideal mix of characteristics. Yes/No questions suffer from a limited range of responses and does not determine how much respondents agree or disagree with a statement. Multiple choice offers more freedom, depending on the context of the question. Descriptive questions make mass administration of the scale difficult, as a lack of standardization means that each response will need to be analyzed; this is challenging for quantitative analysis, although it provides rich qualitative data in small samples.

In terms of the number of points on a Likert scale, measures ranged from 3 to 11. A higher number of options for participants to most accurately represent how they feel may be particularly useful for tracking changes in online wellbeing over time, capturing smaller increases or decreases in mood: an increase from three to four on a 5-point scale is a greater increase than three to four on a 10-point scale, so respondents may be more likely to stick to the lower number. On the other hand, longer scale items may provide too much choice or take longer for respondents to complete, leading to less consistency between answers measuring the same thing: if four or five are seen as very close together, the choice between them may be random and differences missed. “The Paradox of Choice” also argues that excessive choice increases difficulty in choosing an option and decreases satisfaction with the choice (Schwartz, 2004). With the vast majority of scales using between 4 and 7 points, and most using 5, it would be recommended that an OWS follow suit by using 5-point Likert or Likert-like scales. This also makes comparisons with existing offline scales more effective.

Most SWB scales use positive scoring, where a high score indicates good wellbeing. This is likely to be easier to understand and more intuitive (higher = better), and in general, people may be more receptive to “rises in wellbeing” than reporting increases in poor wellbeing. This would suggest that an OWS might be better scored positively; however, in some contexts, this might not make sense. For example, if a questionnaire is asking how often something has occurred, for example, feeling happy or sad, it might be more intuitive for higher scores to equal more often, whether or not the statement was positive. For scales that measure several components of SWB (see next section), having more often high scores for positive items and low for negative items would be confusing. This leads to discussion of the possibility of reverse scoring. The majority of scales did not use reverse scoring. Equal numbers of scales used reverse scoring for only positive or only negative affect items. If positive scoring is used, negative affect questions can be reverse scored; otherwise, a high negative affect score would be taken to mean good wellbeing. There is a theoretical risk that including a high number of reverse-scored questions could increase the complexity; having a mix of positive and negative statements may also cause mistakes from misreading. This has been demonstrated in (Sonderen et al., 2013), which found that reversing the words and hence scoring led to increased respondent inattention and confusion. However, an OWS using positive scoring for the most part and using reverse scoring if negative affect is to be measured along with other SWB components is suggested.

### Subjective Wellbeing Measurements

There is a great amount of variety among scales in terms of which components of SWB they measure. Two-thirds of the scales measured only one component: positive affect, negative affect, or life satisfaction, rather than combining categories. One explanation for this is that question-order effects could be avoided to an extent if all questions are measuring the same thing. However, measuring only one component of SWB neglects the other two and may therefore not be a valid measure of SWB. Measuring both positive and negative affect together was most common among the scales, more than any single component on its own. If (Diener and Suh, 1997) definition of SWB is to be adhered to, positive affect, negative affect, and life satisfaction should all be measured by a scale. However, measuring all components occurred in only two scales.

It is not clear why this is the case; however, there are a few possible explanations. The variety of combinations of components may reflect different aims for the scales; for example, the WEMWBS justifies its focus on positive affect stating that it “supports mental health promotion initiatives” (Tennant et al., 2007, p. 2), whereas the PANAS states that a two-factor model involving both positive and negative affect has “been used more extensively in the self- report mood literature” (Watson et al., 1988, p. 1063). Given the infinite combinations of aims or justifications possible, it should be no surprise that a widespread of SWB components are measured. Furthermore, given that a standardized definition of SWB does not exist (Dodge et al., 2012), the focus of a scale and what aspects of SWB it measures could change depending on how SWB is defined.

In terms of an OWS, some factors usually included in SWB investigations may not be relevant, so it may be that a separate concept of SWB could be adapted for online wellbeing purposes, which will affect the components of wellbeing measured. Based on this review, it is recommended that an OWS at least includes measures of both positive and negative affect, and ideally life satisfaction, with the view to adapting as more research is available. This recommendation is based theoretically on Diener's SWB definition and on the finding that 50% of all scales found used positive affect or negative affect, and 28.9% of all scales used both. In using multiple components, researchers should be aware of potential question-order effects; for example, asking five negative affect questions followed by one positive affect question may negatively prime participants and cause them to rate their positive affect lower than they otherwise would.

### Evaluation Methods

The majority of scales specifies time periods for responses, such as “the past two weeks,” and under one-third used global assessments such as “in general.” Using global assessments without a specified time period could lead to recency bias. If a respondent is asked a global question, the underlying assumption is that they will consider all relevant factors across their entire life and formulate an answer. Instead, research suggests that respondents provide an answer once enough information comes to mind to answer the question with enough certainty that is comfortable for the respondent. Often, this means calling to mind information that was most accessible, which tends to be dependent on the information's frequency of use and recency (Schwarz and Strack, 1999). Additionally, without a specified time period for evaluation, individuals may construct their own time period at the point of asking. For example, one individual may recall information from the past year, whereas another individual may retrieve information from the past month. This could lead to variations between individuals, and within individuals if they are asked at a different time, reducing the external reliability of a scale as a result.

This then raises the question of what time period should be used for an OWS? Using a time window that is too long is problematic, as it risks giving respondents too much room in which to retrieve information for their answer and essentially having the same problems as global assessment. At the other extreme, methods such as the Experience Sampling Method (ESM) (Larson and Csikszentmihalyi, 2014) require respondents to be reminded (through methods such as a buzzer) several times a day to stop and record details such as what they were doing and how they felt according to a questionnaire created by the researcher—an Experience Sampling Form (ESF). However, Alexandrova (2005) argued that such methods were problematic, for example, because insufficient time is given for a respondent to experience the “true” SWB of an event in the long term. Most of the evaluated scales that specified time periods used up to 2 weeks, with very few longer than a month, and this is the recommendation for an OWS. Another option is to suggest various time periods the scale can be used for and let the researcher decide; the PANAS suggests wording for anything from the present moment to in general (Watson et al., 1988).

The vast majority of scales measured wellbeing by directly asking for a subjective evaluation from respondents, with some of these using additional alternative measures. Very few scales only rely on alternative measures; an example is the laughter frequency scale (Murakami et al., 2018), which measures SWB by asking how frequently respondents laughed. SWB scales appear to place emphasis on personal subjective evaluations, which is supported by research into SWB in general (Diener et al., 2018). One study compared scores of the Sakomoto index, a wellbeing index that uses “objective indicators” such as job separation rates (workers becoming unemployed) to rank areas in Japan from most to least “happy.” The study found a negative correlation between self-reports of happiness and the Sakomoto Index (Kuroki, 2013), suggesting flaws in attempts to measure SWB using alternative measures. The issue with alternative measures is that they sometimes make broad assumptions; for example, just because someone reports laughing frequently does not mean that they have an overall high degree of SWB—the person could have other moments of intense sadness. SWB by its nature is subjective; it is critical that a person decides for themselves what is important to them. For these reasons, it is recommended that an OWS mainly focuses on personal subjective evaluations of wellbeing.

However, the notion that objective indicators are not useful and do not correlate to SWB is not true (Oswald and Wu, 2010; Diener et al., 2018). Alternative measures used in conjunction with subjective evaluations can help solve some of the methodological issues surrounding self-reported measures (Diener et al., 2018). For example, contextual factors like the weather at the time of asking may affect someone's SWB evaluation (Schwarz and Clore, 1983). An additional problem of relying solely on subjective responses is that respondents may aggregate their affective responses to an experience in an illogical way, focusing only on intense moments of positive or negative affect and neglecting the overall duration of an experience (Fredrickson and Kahneman, 1993). Thus, judgement on how positive or negative an overall experience is might be skewed by extremes. However, there is no “wrong” way for people to aggregate their affective responses to an experience. For instance, if someone with an otherwise pleasant life has a major traumatic event and rates their overall SWB as fairly negative as a result, it would be unfair to judge the wellbeing evaluation as “wrong.” Given that SWB is what the person says it is, one has the right to judge the traumatic event to be extremely relevant when evaluating their own SWB. There have also been much smaller effect sizes of contextual factors on life satisfaction evaluations (Lucas and Lawless, 2013; Diener et al., 2018). Therefore, contextual alternative measures may be used to complement a predominantly subjective OWS.

Alternative measures should also be phrased in a way that accommodates for different individual ideals. For example, instead of asking “*how much social interaction do you receive?*,” a potentially better question to ask is, “*do you receive enough social interaction to satisfy you?*.” The advantage of the latter is that it accommodates for people who prefer not to interact much with others, and the question asks respondents to compare what they currently have relative to their ideal.

### Limitations

The search for wellbeing scales was intended to be comprehensive but focused, but some literature will be missed, and therefore, there are SWB scales that are not included. To consider more wellbeing scales is outside of this review's aims but is certainly an area for further exploration. Far more wellbeing scales exist, and reading is available such as Boyle et al. (2015). Another issue is that some scales were not available to the authors or were not provided with the scoring system. Gray literature was not included, and databases were searched by titles only, potentially missing out on articles that contained keywords in abstracts or keywords section. Articles only from peer-reviewed journals were included, leading to a potential publication bias. Additionally, papers that refer to SWB only using other words such as “hedonism” or “happiness” will have been missed, as well as other studies that happened to omit the terms “wellbeing” or “scales” and its synonyms. Furthermore, the literature search only searched titles. Future searches may wish to consider including searching by keywords in order to broaden the search and find additional scales. However, as a preliminary scoping review, it provides a broad snapshot of the literature and potential gaps in the research.

Studies were not appraised for quality, in favor of identifying as many scales as possible and not looking into the research carried out with them. Some scales reviewed may be low quality or biased, which could in turn bias the results and conclusion of this paper. However, the vast majority of the scales had some form of validation reported, and only three of the scales were produced solely for the purpose of a single study. Additionally, many of the recommendations were made based on several scales, including many that are strongly validated in previous research. This study was also not designed to determine the reasoning behind design choices in SWB scales, focusing on observing trends in current scales instead. Scoping reviews are intended to be a narrative overview of the literature (Arksey and O'Malley, 2005; Pham et al., 2014); follow-up work may produce more detailed recommendations based on usability and design.

Another limitation is that without a standardized definition of SBW, it can be problematic to recommend existing SWB measurements to the development of a new OWB assessment tool, especially when assuming that the psychometric qualities transferred from SBW to OWB remain the same. This is a limitation that should be kept in mind when applying the recommendations derived from this scoping review.

Finally, one researcher performed the analysis using agreed definitions for terms related to SWB, which could lead to mistakes or biased judgments. However, topic experts were able to agree on stringently defined terms before analysis took place, so it is hoped that another researcher using the same definitions would arrive at similar results. Regardless, multiple researchers analyzing data independently are still preferred.

## Conclusions

This paper describes a broad scoping review of existing measures of SWB, highlighting the lack of online wellbeing measurement research, and compiling features of existing scales to offer recommendations for an OWS. Online wellbeing should be distinguished from generalized SWB, with an OWS focusing on areas of wellbeing that are contextually important to the online world. To summarize, an OWS is recommended to be between 3 and 20 questions and consider developing a brief measure if longer; use mainly 5-point Likert or Likert-like scales, with predominantly positive scoring, using reverse scoring if negative affect is to be measured alongside other SWB components; measure at least positive and negative affect and ideally life satisfaction; ask for evaluations within 1–2 weeks and a month at the maximum; and use mainly subjective evaluations with alternative measures being used as supporting questions if needed.

The search and proposed recommendations for building an OWS are based on how current SWB scales are used in research. By focusing on field-tested SWB scales, the paper examined what is used in practice instead of what works in theory. It needs to be stressed that there is much research to be done to test these recommendations, especially in assessing the accepted components of SWB and how the online world might be unique. Most critically, a clear, accepted definition of the “online world” and what it means in relation to online wellbeing needs to be developed for research into online wellbeing to fruitfully continue. We welcome future work that incorporates the recommendations from this paper to develop and validate a new measure for OWS alongside existing SWB measures to test the assumptions surrounding what effects a persons' online wellbeing.

## Author Contributions

ZO carried out the data collection and analysis and drafted the first version of the paper. LD edited and formatted all drafts of the paper. EP acted as main supervisor for the study, with LD, MR, and YL providing advice at various stages. EP, MR, and YL also commented on versions of the paper and approved the final manuscript. All authors contributed to the article and approved the submitted version.

## Conflict of Interest

The authors declare that the research was conducted in the absence of any commercial or financial relationships that could be construed as a potential conflict of interest.
