# Supplementary material for: Measuring Online Wellbeing: A Scoping Review of Subjective Wellbeing Measures
Source: Front Psychol. 2021 Mar 11;12:616637. doi: 10.3389/fpsyg.2021.616637 (PMC8006413; doi:10.3389/fpsyg.2021.616637)
Supplement: Supplementary file 1 [file Table_1.docx]

Supplementary Material

# Full list of included and excluded scales

- indicates that information is not found

| **Reasons for Exclusion:** | **Types of question** | **Scoring** |
| --- | --- | --- |
| EWB Eudaimonic wellbeing  H Measures health MH Measures mental health  OWB Other wellbeing measure  SNF Scale Not Found | Des Descriptive  n-L n-point Likert scale  Y/N Yes/No questions  MC Multiple Choice questions  var variable question types | pos High score indicates good wellbeing  neg High score indicates poor wellbeing  none No scoring system used  var variable scoring methods  rev Some items reversed scored  No rev items not reversed scored |
| **Subjective wellbeing components:** | **Measures** |  |
| DEP Depression/anxiety symptoms  LS Life Satisfaction  NA Negative Affect  PA Positive Affect  var variable components | Sub Uses subjective measures only  Alt Uses alternative only  Both Uses both alternative and subjective measures  var variable measures |  |

| **Scale** | | **Inclusion** | **Studies using scale** | **Year published** | **Example Reference** | **Number of questions** | **Types of questions** | **Scoring** | **SWB components** | **Timescale** | **Measures** |
| --- | --- | --- | --- | --- | --- | --- | --- | --- | --- | --- | --- |
|  | Age UK Index of Wellbeing in Later Life | Exclude: SNF | 1 | - | (Green et al., n.d.) | - | - | - | - | - | - |
| ATUS:WBM | American Time Use Survey: Wellbeing Module | Include | 3 | 2014 | (Stone et al., 2018) | 10 | 6-L, 10-L | - | PA, NA | Variable | Sub |
| TERTTU | Asylum Seekers Health and Wellbeing Survey | Exclude: MH | 1 | 2019 | (Skogberg et al., 2019) | - | - | - | - | - | - |
| CIW | Canadian Index of Wellbeing | Exclude: OWB | 1 | 2012 | (Muhajarine et al., 2012) | - | - | - | - | - | - |
|  | Cantril Self-Anchoring Scale | Include | 3 | 1966 | (Cantril, 1966) | 10 | Des; 10-L | Pos, no rev | PA, NA | General Future; 5 years ago; 5 years future | Sub |
| CWBI | Caregiver Wellbeing Index | Exclude: SNF | 1 | 2018 | (Betini et al., 2018) | - | - | - | - | - | - |
| CES-D | Centre for Epidemiological Studies Depression Scale | Include | 1 | 1977 | (Radloff, 2016) | 20 | 4-L | neg, rev | DEP | Past week | Sub |
| CES-D8 | Centre for Epidemiological Studies Depression Scale - 8 item version | Include | 1 | 2014 | (Missinne et al., 2014) | 8 | 4-L | neg, rev | DEP | Past week | Both |
| CASP-12 | Control, Autonomy, Self-realization and Pleasure | Exclude: EWB | 2 | 2003 | (Hyde et al., 2003) | - | - | - | - | - | - |
| DWB | Daily Wellbeing Questionnaire | Include | 1 | 2010 | (McLean et al., 2010) | 4 | 5-L | Pos, no rev | PA, NA | General | Both |
| DRM | Day Reconstruction Method | Include | 1 | 2004 | (Kahneman et al., 2004) | 12 | 7-L | -, no rev | PA, NA | Past day | Sub |
| ERQ | Emotion Regulation Questionnaire | Include | 2 | 2003 | (Gross & John, 2003) | 10 | 7-L | none, no rev | PA | General | Both |
| EQ-5D-3L | EuroQoL – 5 Dimensions – 3 Levels | Include | 1 | 1990 | (EuroQol Group, 1990) | 5 | MC | var, no rev | NA | Today | Both |
| EQ-VAS | EuroQoL-Visual Analogue Scales | Exclude: H | 1 | 1990 | (EuroQol Group, 1990) | - | - | - | - | - | - |
| ESS | European Social Survey: Wellbeing Module | Include | 1 | 2009 | (Huppert et al., 2009) | 36 | 4-L, 5-L, 6-L, 7-L, 11-L | -, - | PA, NA, LS | General, past week | Both |
| ESF | Experience Sampling form, derived from Experience Sampling Method (ESM) | Include | 1 | 1983 | (Larson & Csikszentmihalyi, 2014) | var | var | var | var | Instant | var |
| FMPS | Frost Multidimensional Perfectionism Scale | Exclude: OWB | 1 | 1998 | (Frost et al., 1990) | - | - | - | - | - | - |
|  | Gallup-Healthways Wellbeing Index | Exclude: SNF | 1 | 2008 | (Gurley, 2008) | - | - | - | - | - | - |
| GHQ-12 | General Health Questionnaire-12 | Include | 5 | 1988 | (Goldberg & Williams, 1988) | 12 | 4-L | var | DEP | Past few weeks | Both |
| SHS | Subjective Happiness Scale | Include | 1 | 1999 | (Lyubomirsky & Lepper, 1999) | 4 | 7-L | pos, rev | PA, NA | General | Sub |
| GLS | Global Life Satisfaction | Include | 4 | 2003 | (Cummins et al., 2003) | 1 | 11-L | pos, no rev | LS | General | Sub |
| HPMood | Homeostatically Protected Mood | Include | 1 | 2018 | (Capic et al., 2018) | 3 | 11-L | Pos, no rev | PA | General | Sub |
|  | Index of Psychological Wellbeing at Work | Exclude: SNF | 1 | 2016 | (Royer & Moreau, 2016) | - | - | - | - | - | - |
|  | Kearns & Whitley Loneliness Scale | Include | 1 | 2019 | (Kearns & Whitley, 2019) | 1 | 3-L | none | NA | Past 2 weeks | Alt |
| K-6 |  | Include | 1 | 2002 | (Kessler et al., 2002) | 6 | 5-L | Pos, no rev | DEP | Past 30 days | Both |
| K-10 |  | Include | 1 | 2003 | (Kessler et al., 2003) | 10 | 5-L | Neg, no rev | DEP | Past 30 days | Sub |
| MIDI | Midlife Development Index | Exclude: EWB | 1 | 1997 | (Brim et al., 2019) | - | - | - | - | - | - |
| MCC | Moores Cancer Centre Wellbeing Screening tool | Include | 1 | 2019 | (Cardenas et al., 2019) | 15 | 4-L | Neg, - | NA | Past week | sub |
| MDMQ | Multi-dimensional Mood State Questionnaire | Include | 1 | 1997 | (R Steyer et al., 2004) | 30 | 6-L | -, - | PA, NA | Instant | Sub |
|  | Murakami Laughter Frequency | Include | 1 | 2018 | (Murakami et al., 2018) | 1 | 4-L | None | PA | General | Alt |
| MWM-OA | Museum Wellbeing Measure – Older Adults | Include | 1 | 2015 | (Thomson & Chatterjee, 2014) | 6 | 5-L | Pos, no rev | PA | General | Sub |
| NWI | National Wellbeing Index | Include | 3 | 2003 | (Cummins et al., 2003) | 22 | 10-L, Y/N, MC | Pos, - | LS | General, in the near future | Sub |
| NLES | Negative Life Experiences Scale | Include | 1 | 2007 | (Kowal et al., 2007) | 16 | Y/N | Neg | NA | Past year | alt |
| PIWBS-R | Pacific Identity and Wellbeing Scale revised | Exclude: SNF | 1 | 2015 | (Manuela & Sibley, 2015) | - | - | - | - | - | - |
| PHQ-4 | Patient Health Questionnaire-4 | Include | 1 | 2009 | (Kroenke et al., 2009) | 4 | 4-L | Neg, no rev | DEP | Past 2 weeks | sub |
| PATS | Princeton Affect and Time Survey | Exclude: SNF | 1 | 2006 | (Kahneman & Krueger, 2006) | - | - | - | - | - | - |
| PSS | Perceived Stress Scale | Include | 4 | 1983 | (Cohen et al., 1983) | 10 | 5-L | Neg, rev | NA | Last month | Sub |
| PWI | Personal Wellbeing Index (subscale of the Australian Unity Wellbeing Index) | Include | 10 | 2003 | (Cummins et al., 2003) | 7 | 10-L | Pos, no rev | LS | General | Sub |
| PANAS | Positive Affect Negative Affect Schedule | Include | 6 | 1988 | (Watson et al., 1988) | 20 | 5-L | var, no rev | PA, NA | Variable | Sub |
| POMS-SF | Profile of Moods States – Short Form | Include | 1 | 1983 | (Curran et al., 1995) | 37 | 5-L | -, no rev | NA | Past week | Sub |
|  | Ryff’s Scales of Psychological Wellbeing | Exclude: EWB | 2 | 1989 | (Ryff, 1989) | - | - | - | - | - | - |
| SWLS | Satisfaction with Life Scale | Include | 1 | 1985 | (Diener & Suh, 1997) | 5 | 7-L | Pos, no rev | LS | General | Sub |
|  | Self-Rated Health | Exclude: H | 1 | - | Not Applicable – simple question on how their health is | - | - | - | - | - | - |
| SWEMWBS | Shortened Warwick-Edinburgh Mental Wellbeing Scale | Include | 2 | 2006 | (Haver et al., 2015) | 7 | 5-L | Pos, rev | PA | Past 2 weeks | Both |
| SPS | Social Provisions Scale | Include | 1 | 1983 | (Cutrona & Russell, 1983) | 24 | 4-L | Pos, rev | NA | General | Alt |
|  | Sørensen et al Questionnaire | Exclude: OWB | 1 | 2016 | (Sørensen et al., 2016) | - | - | - | - | - | - |
|  | Spiritual Wellbeing Scale | Exclude: SNF | 1 | 1991 | (Bufford et al., 1991) | - | - | - | - | - | - |
|  | St Elizabeth Youngstown Hospital Wellbeing Inventory | Include | 1 | 2019 | (Dunham et al., 2019) | 10 | 5-L | -,- | PA, NA | Past 3 days | Both |
| TSWQ | Teacher Subjective Wellbeing Questionnaire | Exclude: OWB | 1 | 2015 | (Renshaw et al., 2015) | - | - | - | - | - | - |
| ULS-R | UCLA Loneliness Scale – Revised | Include | 1 | 1980 | (D. Russell et al., 1980) | 20 | 4-L | Neg, rev | NA | General | Alt |
|  | UK ONS Wellbeing Scale | Include | 1 | 2018 | (Office for National Statistics, 2018) | 4 | 11-L | Var, no rev | PA, LS, DEP | General, yesterday | Sub |
| UMACL | UWIST Mood Adjective Checklist | Include | 1 | 1990 | (Matthews et al., 1990) | 29 | 4-L | Var, - | PA, NA | Instant | Sub |
| WEMWBS | Warwick-Edinburgh Mental Wellbeing Scale | Include | 12 | 2007 | (Tennant et al., 2007) | 14 | 5-L | Pos, rev | PA | Past 2 weeks | Both |
| WHOQoL-BREF | WHO Quality of Life – Brief | Include | 1 | 1996 | (WHO, 1996) | 26 | 5-L | Pos, no rev | PA, NA, LS | General, past 2 weeks | Both |
| WHO-5 | WHO-5 Wellbeing Index | Include | 5 | 2015 | (Topp et al., 2015) | 5 | 6-L | Pos, no rev | PA | Past 2 weeks | Sub |

# Validation and evaluation status of included scales

| **Scale** | | **Paper describing creation** | **Reference for paper describing wellbeing measure development** | **Has a 3^rd^ party evaluated the scale?** | **Reference(s) for 3^rd^ party evaluation** |
| --- | --- | --- | --- | --- | --- |
| ATUS:WBM | American Time Use Survey: Wellbeing Module | Yes | (Horrigan & Herz, 2004) | Yes | (Y. Lee et al., 2016) |
|  | Cantril Self-Anchoring Scale | Yes | (Cantril, 1966) | Not Found | - |
| CES-D | Centre for Epidemiological Studies Depression Scale | Yes | (Radloff, 1977) | Yes | (Milette et al., 2010; Roberts, 1980) |
| CES-D8 | Centre for Epidemiological Studies Depression Scale - 8 item version | Yes | (Radloff, 1977) | Yes | (O’Halloran et al., 2014) |
| DWB | Daily Wellbeing Questionnaire | Not found | - | Not found | - |
| DRM | Day Reconstruction Method | Yes | (Kahneman et al., 2004) | Yes | (Ayuso-Mateos et al., 2013; Diener & Tay, 2014; Lucas et al., 2020) |
| ERQ | Emotion Regulation Questionnaire | Yes | (Gross & John, 2003) | Yes | (Preece et al., 2020) |
| EQ-5D-3L | EuroQoL – 5 Dimensions – 3 Levels | Guidance not paper | (EuroQol Group, 1990) | Yes | (Janssen et al., 2013) |
| ESS | European Social Survey: Wellbeing Module | Yes | (Huppert et al., 2009) | Not Found | - |
| ESF | Experience Sampling form, derived from Experience Sampling Method (ESM) | Book Published | (Larson & Csikszentmihalyi, 2014) | Yes | (Csikszentmihalyi & Larson, 2014; Lucas et al., 2020) |
| GHQ-12 | General Health Questionnaire-12 | Guidance not paper | (Goldberg & Williams, 1988) | Yes | (Smith et al., 2010) |
| SHS | Subjective Happiness Scale | Yes | (Lyubomirsky & Lepper, 1999) | Not Found | - |
| GLS | Global Life Satisfaction | Yes | (Cummins et al., 2003) | Not Found | - |
| HPMood | Homeostatically Protected Mood | Yes | (Capic et al., 2018) | Not Found | - |
|  | Kearns & Whitley Loneliness Scale | Not found | - | Not Found | - |
| K-6 |  | Yes | (Ronald C. Kessler et al., 2003) | Not Found | - |
| K-10 |  | Yes | (R. C. Kessler et al., 2002) | Not Evaluated for Subjective Wellbeing | - |
| MCC | Moores Cancer Centre Wellbeing Screening tool | Yes | (Cardenas et al., 2019) | Not Found | - |
| MDMQ | Multi-dimensional Mood State Questionnaire | Yes | (Rolf Steyer et al., 1997) | Not Found | - |
|  | Murakami Laughter Frequency | Not found | - | Not Found | - |
| MWM-OA | Museum Wellbeing Measure – Older Adults | Yes | (Thomson & Chatterjee, 2015) | Not Found | - |
| NWI | National Wellbeing Index | Yes | (Cummins et al., 2003) | Not Found | - |
| NLES | Negative Life Experiences Scale | Yes | (Kowal et al., 2007) | Not Found | - |
| PHQ-4 | Patient Health Questionnaire-4 | Yes | (Kroenke et al., 2009) | Not Evaluated for Subjective Wellbeing | - |
| PSS | Perceived Stress Scale | Yes | (Cohen et al., 1983) | Yes | (B. Lee & Jeong, 2019; Teresi et al., 2020) |
| PWI | Personal Wellbeing Index (subscale of the Australian Unity Wellbeing Index) | Yes | (Cummins et al., 2003) | Not found | - |
| PANAS | Positive Affect Negative Affect Schedule | Yes | (Watson et al., 1988) | Yes | (Crawford & Henry, 2004) |
| POMS-SF | Profile of Moods States – Short Form | Yes | (Curran et al., 1995) | Not Found | - |
| SWLS | Satisfaction with Life Scale | Yes | (Diener et al., 1985) | Not Found published in peer-reviewed journal | - |
| SWEMWBS | Shortened Warwick-Edinburgh Mental Wellbeing Scale | Yes | (Haver et al., 2015) | Yes | (Blenkiron & Goldsmith, 2019; Koushede et al., 2019; Shah et al., 2018) |
| SPS | Social Provisions Scale | Yes | (Cutrona & Russell, 1983) | Yes | (Zaki, 2009) |
|  | St Elizabeth Youngstown Hospital Wellbeing Inventory | Yes | (Dunham et al., 2019) | Not Found | - |
| ULS-R | UCLA Loneliness Scale – Revised | Yes | (Dan Russell et al., 1980) | Not Found | - |
|  | UK ONS Wellbeing Scale | Guidance not paper | (Office for National Statistics, 2018) | Not Found | - |
| UMACL | UWIST Mood Adjective Checklist | Yes | (Matthews et al., 1990) | Not Found | - |
| WEMWBS | Warwick-Edinburgh Mental Wellbeing Scale | Yes | (Tennant et al., 2007) | Yes | (Maheswaran et al., 2012; Stewart-Brown et al., 2011; Taggart et al., 2013) |
| WHOQoL-BREF | WHO Quality of Life – Brief | Guidance not paper | (WHO, 1996) | Not Found | (Duarte et al., 2020; Pomeroy et al., 2020; Young et al., 2020) |
| WHO-5 | WHO-5 Wellbeing Index | Yes | (Topp et al., 2015) | Yes | (Primack, 2003) |

**3. Full references for scales in Supplementary Material sections 1 and 2**

Ayuso-Mateos, J. L., Miret, M., Caballero, F. F., Olaya, B., Haro, J. M., Kowal, P., & Chatterji, S. (2013). Multi-Country Evaluation of Affective Experience: Validation of an Abbreviated Version of the Day Reconstruction Method in Seven Countries. *PLOS ONE*, *8*(4), e61534. https://doi.org/10.1371/journal.pone.0061534

Betini, R. S. D., Hirdes, J. P., Curtin-Telegdi, N., Gammage, L., Vansickle, J., Poss, J., & Heckman, G. (2018). Development and validation of a screener based on interRAI assessments to measure informal caregiver wellbeing in the community. *BMC Geriatrics*, *18*(1), 310. https://doi.org/10.1186/s12877-018-0986-x

Blenkiron, P., & Goldsmith, L. (2019). Patient-reported outcome measures in community mental health teams: Pragmatic evaluation of PHQ-9, GAD-7 and SWEMWBS. *BJPsych Bulletin*, *43*(5), 221–227. https://doi.org/10.1192/bjb.2019.20

Brim, O. G., Ryff, C. D., & Kessler, R. C. (2019). *How Healthy Are We?: A National Study of Well-Being at Midlife*. University of Chicago Press.

Bufford, R. K., Paloutzian, R. F., & Ellison, C. W. (1991). Norms for the Spiritual Weil-Being Scale. *Journal of Psychology and Theology*, *19*(1), 56–70. https://doi.org/10.1177/009164719101900106

Cantril, H. (1966). *The pattern of human concerns.* Rutgers University Press.

Capic, T., Li, N., & Cummins, R. A. (2018). Confirmation of Subjective Wellbeing Set-Points: Foundational for Subjective Social Indicators. *Social Indicators Research: An International and Interdisciplinary Journal for Quality-of-Life Measurement*, *137*(1), 1–28.

Cardenas, V., Abbott, Y., Hirst, J. M., Mausbach, B. T., Agarwal, S., Collier, G., Tran, L., Tamayo, G., Downey, A., Herring, M., & Irwin, S. A. (2019). Development, implementation, and initial results of the UC San Diego Health Moores Cancer Center Wellbeing Screening Tool. *Palliative & Supportive Care*, *17*(4), 431–435. https://doi.org/10.1017/S1478951518000810

Cohen, S., Kamarck, T., & Mermelstein, R. (1983). A global measure of perceived stress. *Journal of Health and Social Behavior*, *24*(4), 385–396.

Crawford, J. R., & Henry, J. D. (2004). The Positive and Negative Affect Schedule (PANAS): Construct validity, measurement properties and normative data in a large non-clinical sample. *British Journal of Clinical Psychology*, *43*(3), 245–265. https://doi.org/10.1348/0144665031752934

Csikszentmihalyi, M., & Larson, R. (2014). Validity and Reliability of the Experience-Sampling Method. *Flow and the Foundations of Positive Psychology: The Collected Works of Mihaly Csikszentmihalyi*, 35–54. https://doi.org/10.1007/978-94-017-9088-8_3

Cummins, R. A., Eckersley, R., Pallant, J., Vugt, J. van, & Misajon, R. (2003). Developing a national index of subjective wellbeing: The Australian Unity Wellbeing Index. *Social Indicators Research*, *64*(2), 159–190.

Curran, S., Andrykowski, M., & Studts, J. (1995). Short Form of the Profile of Mood States (POMS–SF): Psychometric Information. *Psychological Assessment*, *7*(1), 80–83. https://doi.org/10.1037/1040-3590.7.1.80

Cutrona, C., & Russell, D. (1983). The Provisions of Social Relationships and Adaptation to Stress. In W. H. Jones & D. Perlman, *Advances in Personal Relationships* (Vol. 1, pp. 37–67). JAI Press.

Diener, E., Emmons, R. A., Larsen, R. J., & Griffin, S. (1985). The Satisfaction With Life Scale. *Journal of Personality Assessment*, *49*(1), 71–75. https://doi.org/10.1207/s15327752jpa4901_13

Diener, E., & Suh, E. (1997). Measuring Quality of Life: Economic, Social, and Subjective Indicators. *Social Indicators Research*, *40*(1), 189–216. https://doi.org/10.1023/A:1006859511756

Diener, E., & Tay, L. (2014). Review of the Day Reconstruction Method (DRM). *Social Indicators Research*, *116*(1), 255–267. https://doi.org/10.1007/s11205-013-0279-x

Duarte, S. B. R., Chaveiro, N., de Freitas, A. R., Barbosa, M. A., Camey, S., Fleck, M. P., Porto, C. C., Rodrigues, C. L., & Rodríguez-Martín, D. (2020). Validation of the WHOQOL-Bref instrument in Brazilian sign language (Libras). *Quality of Life Research*. https://doi.org/10.1007/s11136-020-02611-5

Dunham, C. M., Burger, A. L., Hileman, B. M., & Chance, E. A. (2019). Psychometric properties of the St. Elizabeth Youngstown hospital wellbeing inventory and non-burnout inventory for physicians and nurses. *BMC Psychology*, *7*(1), 36. https://doi.org/10.1186/s40359-019-0316-x

EuroQol Group. (1990). EuroQol—A new facility for the measurement of health-related quality of life. *Health Policy (Amsterdam, Netherlands)*, *16*(3), 199–208. https://doi.org/10.1016/0168-8510(90)90421-9

Frost, R. O., Marten, P., Lahart, C., & Rosenblate, R. (1990). The dimensions of perfectionism. *Cognitive Therapy and Research*, *14*(5), 449–468. https://doi.org/10.1007/BF01172967

Goldberg, D. P., & Williams, D. P. M., Paul. (1988). *A user’s guide to the General Health Questionnaire*. Windsor, Berks. : NFER-Nelson. https://trove.nla.gov.au/version/21182576

Green, M., Iparraguirre, J., Davidson, S., Rossall, P., Ray, S., & Zaidi, A. (n.d.). *Methodology of Age UK’s Index of Wellbeing in Later Life*. Age UK.

Gross, J. J., & John, O. P. (2003). Individual differences in two emotion regulation processes: Implications for affect, relationships, and well-being. *Journal of Personality and Social Psychology*, *85*(2), 348–362. https://doi.org/10.1037/0022-3514.85.2.348

Gurley, J. K. H. and V. F. (2008). Measuring Well-Being in the United States. *APS Observer*, *21*(8). https://www.psychologicalscience.org/observer/measuring-well-being-in-the-united-states

Haver, A., Akerjordet, K., Caputi, P., Furunes, T., & Magee, C. (2015). Measuring mental well-being: A validation of the Short Warwick-Edinburgh Mental Well-Being Scale in Norwegian and Swedish. *Scandinavian Journal of Public Health*, *43*(7), 721–727. https://doi.org/10.1177/1403494815588862

Horrigan, M., & Herz, D. (2004). A Study in the Process of Planning, Designing and Executing a Survey Program: The BLS American Time-Use Survey. In *Contributions to Economic Analysis* (Vol. 271, pp. 317–350). Elsevier. https://doi.org/10.1016/S0573-8555(04)71012-3

Huppert, F. A., Marks, N., Clark, A., Siegrist, J., Stutzer, A., Vittersø, J., & Wahrendorf, M. (2009). Measuring Well-being Across Europe: Description of the ESS Well-being Module and Preliminary Findings. *Social Indicators Research*, *91*(3), 301–315. https://doi.org/10.1007/s11205-008-9346-0

Hyde, M., Wiggins, R. D., Higgs, P., & Blane, D. B. (2003). A measure of quality of life in early old age: The theory, development and properties of a needs satisfaction model (CASP-19). *Aging & Mental Health*, *7*(3), 186–194. https://doi.org/10.1080/1360786031000101157

Janssen, M. F., Pickard, A. S., Golicki, D., Gudex, C., Niewada, M., Scalone, L., Swinburn, P., & Busschbach, J. (2013). Measurement properties of the EQ-5D-5L compared to the EQ-5D-3L across eight patient groups: A multi-country study. *Quality of Life Research*, *22*(7), 1717–1727. https://doi.org/10.1007/s11136-012-0322-4

Kahneman, D., & Krueger, A. B. (2006). Developments in the Measurement of Subjective Well-Being. *Journal of Economic Perspectives*, *20*(1), 3–24. https://doi.org/10.1257/089533006776526030

Kahneman, D., Krueger, A. B., Schkade, D. A., Schwarz, N., & Stone, A. A. (2004). A survey method for characterizing daily life experience: The day reconstruction method. *Science (New York, N.Y.)*, *306*(5702), 1776–1780. https://doi.org/10.1126/science.1103572

Kearns, A., & Whitley, E. (2019). Associations of internet access with social integration, wellbeing and physical activity among adults in deprived communities: Evidence from a household survey. *BMC Public Health*, *19*(1), 860. https://doi.org/10.1186/s12889-019-7199-x

Kessler, R. C., Andrews, G., Colpe, L. J., Hiripi, E., Mroczek, D. K., Normand, S. L. T., Walters, E. E., & Zaslavsky, A. M. (2002). Short screening scales to monitor population prevalences and trends in non-specific psychological distress. *Psychological Medicine*, *32*(6), 959–976. https://doi.org/10.1017/s0033291702006074

Kessler, Ronald C., Barker, P. R., Colpe, L. J., Epstein, J. F., Gfroerer, J. C., Hiripi, E., Howes, M. J., Normand, S.-L. T., Manderscheid, R. W., Walters, E. E., & Zaslavsky, A. M. (2003). Screening for serious mental illness in the general population. *Archives of General Psychiatry*, *60*(2), 184–189. https://doi.org/10.1001/archpsyc.60.2.184

Koushede, V., Lasgaard, M., Hinrichsen, C., Meilstrup, C., Nielsen, L., Rayce, S. B., Torres-Sahli, M., Gudmundsdottir, D. G., Stewart-Brown, S., & Santini, Z. I. (2019). Measuring mental well-being in Denmark: Validation of the original and short version of the Warwick-Edinburgh mental well-being scale (WEMWBS and SWEMWBS) and cross-cultural comparison across four European settings. *Psychiatry Research*, *271*, 502–509. https://doi.org/10.1016/j.psychres.2018.12.003

Kowal, E., Gunthorpe, W., & Bailie, R. S. (2007). Measuring emotional and social wellbeing in Aboriginal and Torres Strait Islander populations: An analysis of a Negative Life Events Scale. *International Journal for Equity in Health*, *6*(1), 18. https://doi.org/10.1186/1475-9276-6-18

Kroenke, K., Spitzer, R. L., Williams, J. B. W., & Löwe, B. (2009). An ultra-brief screening scale for anxiety and depression: The PHQ-4. *Psychosomatics*, *50*(6), 613–621. https://doi.org/10.1176/appi.psy.50.6.613

Larson, R., & Csikszentmihalyi, M. (2014). The Experienec Sampling Method. In *Flow and the Foundations of Positive Psychology: The Collected Works of Mihaly Csikszentmihalyi* (pp. 21–34). Springer Netherlands. https://doi.org/10.1007/978-94-017-9088-8_2

Lee, B., & Jeong, H. I. (2019). Construct validity of the perceived stress scale (PSS-10) in a sample of early childhood teacher candidates. *Psychiatry and Clinical Psychopharmacology*, *29*(1), 76–82. https://doi.org/10.1080/24750573.2019.1565693

Lee, Y., Hofferth, S. L., Flood, S. M., & Fisher, K. (2016). Reliability, Validity, and Variability of the Subjective Well-Being Questions in the 2010 American Time Use Survey. *Social Indicators Research*, *126*(3), 1355–1373. https://doi.org/10.1007/s11205-015-0923-8

Lucas, R. E., Wallsworth, C., Anusic, I., & Donnellan, M. B. (2020). A direct comparison of the day reconstruction method (DRM) and the experience sampling method (ESM). *Journal of Personality and Social Psychology*, No Pagination Specified-No Pagination Specified. https://doi.org/10.1037/pspp0000289

Lyubomirsky, S., & Lepper, H. S. (1999). A measure of subjective happiness: Preliminary reliability and construct validity. *Social Indicators Research*, *46*(2), 137–155.

Maheswaran, H., Weich, S., Powell, J., & Stewart-Brown, S. (2012). Evaluating the responsiveness of the Warwick Edinburgh Mental Well-Being Scale (WEMWBS): Group and individual level analysis. *Health and Quality of Life Outcomes*, *10*(1), 156. https://doi.org/10.1186/1477-7525-10-156

Manuela, S., & Sibley, C. G. (2015). The Pacific Identity and Wellbeing Scale-Revised (PIWBS-R). *Cultural Diversity & Ethnic Minority Psychology*, *21*(1), 146–155. https://doi.org/10.1037/a0037536

Matthews, G., Jones, D. M., & Chamberlain, A. G. (1990). Refining the measurement of mood: The UWIST Mood Adjective Checklist. *British Journal of Psychology*, *81*(1), 17–42. https://doi.org/10.1111/j.2044-8295.1990.tb02343.x

McLean, B. D., Coutts, A. J., Kelly, V., McGuigan, M. R., & Cormack, S. J. (2010). Neuromuscular, Endocrine, and Perceptual Fatigue Responses During Different Length Between-Match Microcycles in Professional Rugby League Players. *International Journal of Sports Physiology and Performance*, *5*(3), 367–383. https://doi.org/10.1123/ijspp.5.3.367

Milette, K., Hudson, M., Baron, M., Thombs, B. D., & Group*, C. S. R. (2010). Comparison of the PHQ-9 and CES-D depression scales in systemic sclerosis: Internal consistency reliability, convergent validity and clinical correlates. *Rheumatology*, *49*(4), 789–796. https://doi.org/10.1093/rheumatology/kep443

Missinne, S., Vandeviver, C., Van de Velde, S., & Bracke, P. (2014). Measurement equivalence of the CES-D 8 depression-scale among the ageing population in eleven European countries. *Social Science Research*, *46*, 38–47. https://doi.org/10.1016/j.ssresearch.2014.02.006

Muhajarine, N., Labonte, R., & Winquist, B. D. (2012). The Canadian Index of Wellbeing: Key findings from the healthy populations domain. *Canadian Journal of Public Health = Revue Canadienne De Sante Publique*, *103*(5), e342-347.

Murakami, M., Hirosaki, M., Suzuki, Y., Maeda, M., Yabe, H., Yasumura, S., & Ohira, T. (2018). Reduction of radiation-related anxiety promoted wellbeing after the 2011 disaster: ‘Fukushima Health Management Survey’. *Journal of Radiological Protection: Official Journal of the Society for Radiological Protection*, *38*(4), 1428–1440. https://doi.org/10.1088/1361-6498/aae65d

Office for National Statistics. (2018). *Personal well-being user guidance*. https://www.ons.gov.uk/peoplepopulationandcommunity/wellbeing/methodologies/personalwellbeingsurveyuserguide

O’Halloran, A. M., Kenny, R. A., & King-Kallimanis, B. L. (2014). The latent factors of depression from the short forms of the CES-D are consistent, reliable and valid in community-living older adults. *European Geriatric Medicine*, *5*(2), 97–102. https://doi.org/10.1016/j.eurger.2013.12.004

Pomeroy, I. M., Tennant, A., Mills, R. J., Young, C. A., & TONiC Study Group. (2020). The WHOQOL-BREF: A modern psychometric evaluation of its internal construct validity in people with multiple sclerosis. *Quality of Life Research*, *29*(7), 1961–1972. https://doi.org/10.1007/s11136-020-02463-z

Preece, D. A., Becerra, R., Robinson, K., & Gross, J. J. (2020). The Emotion Regulation Questionnaire: Psychometric Properties in General Community Samples. *Journal of Personality Assessment*, *102*(3), 348–356. https://doi.org/10.1080/00223891.2018.1564319

Primack, B. (2003). The WHO-5 Wellbeing index performed the best in screening for depression in primary care. *ACP Journal Club*. https://doi.org/10.1136/ebm.8.5.155

Radloff, L. S. (1977). The CES-D Scale: A Self-Report Depression Scale for Research in the General Population. *Applied Psychological Measurement*, *1*(3), 385–401. https://doi.org/10.1177/014662167700100306

Radloff, L. S. (2016). The CES-D Scale: A Self-Report Depression Scale for Research in the General Population. *Applied Psychological Measurement*. https://doi.org/10.1177/014662167700100306

Renshaw, T. L., Long, A. C. J., & Cook, C. R. (2015). Assessing teachers’ positive psychological functioning at work: Development and validation of the Teacher Subjective Wellbeing Questionnaire. *School Psychology Quarterly: The Official Journal of the Division of School Psychology, American Psychological Association*, *30*(2), 289–306. https://doi.org/10.1037/spq0000112

Roberts, R. E. (1980). Reliability of the CES-D scale in different ethnic contexts. *Psychiatry Research*, *2*(2), 125–134. https://doi.org/10.1016/0165-1781(80)90069-4

Royer, N., & Moreau, C. (2016). A Survey of Canadian Early Childhood Educators’ Psychological Wellbeing at Work. *Early Childhood Education Journal*, *44*(2), 135–146. https://doi.org/10.1007/s10643-015-0696-3

Russell, D., Peplau, L. A., & Cutrona, C. E. (1980). The revised UCLA Loneliness Scale: Concurrent and discriminant validity evidence. *Journal of Personality and Social Psychology*, *39*(3), 472–480. https://doi.org/10.1037//0022-3514.39.3.472

Russell, Dan, Peplau, L. A., & Cutrona, C. E. (1980). The Revised UCLA Loneliness Scale: Concurrent and Discriminant Validity Evidence. *Journal of Personality and Social Psychology*, *39*(3), 472.

Ryff, C. D. (1989). Happiness Is Everything, or Is It? Explorations on the Meaning of Psychological Well-Being. *Journal of Personality and Social Psychology*, *57*(6), 1069–1081.

Shah, N., Cader, M., Andrews, W. P., Wijesekera, D., & Stewart-Brown, S. L. (2018). Responsiveness of the Short Warwick Edinburgh Mental Well-Being Scale (SWEMWBS): Evaluation a clinical sample. *Health and Quality of Life Outcomes*, *16*(1), 239. https://doi.org/10.1186/s12955-018-1060-2

Skogberg, N., Koponen, P., Tiittala, P., Mustonen, K.-L., Lilja, E., Snellman, O., & Castaneda, A. (2019). Asylum seekers health and wellbeing (TERTTU) survey: Study protocol for a prospective total population health examination survey on the health and service needs of newly arrived asylum seekers in Finland. *BMJ Open*, *9*(4), e027917. https://doi.org/10.1136/bmjopen-2018-027917

Smith, A. B., Fallowfield, L. J., Stark, D. P., Velikova, G., & Jenkins, V. (2010). A Rasch and confirmatory factor analysis of the General Health Questionnaire (GHQ)—12. *Health and Quality of Life Outcomes*, *8*(1), 45. https://doi.org/10.1186/1477-7525-8-45

Sørensen, N. N., Løje, H., Tetens, I., Wu, J. H. Y., Neal, B., & Lassen, A. D. (2016). Wellbeing at work among kitchen workers during organic food conversion in Danish public kitchens: A longitudinal survey. *The European Journal of Public Health*, *26*(2), 323–328. https://doi.org/10.1093/eurpub/ckv229

Stewart-Brown, S. L., Platt, S., Tennant, A., Maheswaran, H., Parkinson, J., Weich, S., Tennant, R., Taggart, F., & Clarke, A. (2011). The Warwick-Edinburgh Mental Well-being Scale (WEMWBS): A valid and reliable tool for measuring mental well-being in diverse populations and projects. *J Epidemiol Community Health*, *65*(Suppl 2), A38–A39. https://doi.org/10.1136/jech.2011.143586.86

Steyer, R, Schwenkmezger, P., Notz, P., & Eid, M. (2004). Development of the multi-dimensional health questionnaire (MDBF). Primary record. (Version 1.0.0) [data and documentation]. *Trier: Psychological Data Archive PsychData of the Leibniz Center for Psychological Information and Documentation ZPID.* https://doi.org/10.5160/psychdata.srrf91en15

Steyer, Rolf, Schwenkmezger, P., Notz, P., & Eid, M. (1997). Der Mehrdimensionale Befindlichkeitsfragebogen MDBF [Multidimensional mood questionnaire]. *Göttingen, Germany: Hogrefe*.

Stone, A. A., Schneider, S., Krueger, A., Schwartz, J. E., & Deaton, A. (2018). Experiential wellbeing data from the American Time Use Survey: Comparisons with other methods and analytic illustrations with age and income. *Social Indicators Research*, *136*(1), 359–378. https://doi.org/10.1007/s11205-016-1532-x

Taggart, F., Friede, T., Weich, S., Clarke, A., Johnson, M., & Stewart-Brown, S. (2013). Cross cultural evaluation of the Warwick-Edinburgh mental well-being scale (WEMWBS) -a mixed methods study. *Health and Quality of Life Outcomes*, *11*(1), 27. https://doi.org/10.1186/1477-7525-11-27

Tennant, R., Hiller, L., Fishwick, R., Platt, S., Joseph, S., Weich, S., Parkinson, J., Secker, J., & Stewart-Brown, S. (2007). The Warwick-Edinburgh Mental Well-being Scale (WEMWBS): Development and UK validation. *Health and Quality of Life Outcomes*, *5*(1), 63. https://doi.org/10.1186/1477-7525-5-63

Teresi, J. A., Ocepek-Welikson, K., Ramirez, M., Kleinman, M., Ornstein, K., Siu, A., & Luchsinger, J. (2020). Evaluation of the measurement properties of the Perceived Stress Scale (PSS) in Hispanic caregivers to patients with Alzheimer’s disease and related disorders. *International Psychogeriatrics*, *32*(9), 1073–1084. https://doi.org/10.1017/S1041610220000502

Thomson, L. J., & Chatterjee, H. J. (2014). Measuring the impact of museum activities on well-being: Developing the Museum Well-being Measures Toolkit. *Museum Management and Curatorship*, *30*(1), 44–62. https://doi.org/10.1080/09647775.2015.1008390

Thomson, L. J., & Chatterjee, H. J. (2015). Measuring the impact of museum activities on well-being: Developing the Museum Well-being Measures Toolkit. *Museum Management and Curatorship*, *30*(1), 44–62. https://doi.org/10.1080/09647775.2015.1008390

Topp, C. W., Østergaard, S. D., Søndergaard, S., & Bech, P. (2015). The WHO-5 Well-Being Index: A systematic review of the literature. *Psychotherapy and Psychosomatics*, *84*(3), 167–176. https://doi.org/10.1159/000376585

Watson, D., Clark, L. A., & Tellegen, A. (1988). Development and Validation of Brief Measures of Positive and Negative Affect: The PANAS scales. *Journal of Personality and Social Psychology*, *54*(6), 1063–1070. https://doi.org/10.1037/0022-3514.54.6.1063

WHO. (1996). *WHOQOL-BREF Introduction, Administration, Scoring, and Generic Version of the Assessment*. World Health Organisation. https://www.who.int/mental_health/media/en/76.pdf

Young, C. A., Mills, R., Al-Chalabi, A., Burke, G., Chandran, S., Dick, D. J., Ealing, J., Hanemann, C. O., Harrower, T., Mcdermott, C. J., Majeed, T., Pinto, A., Talbot, K., Walsh, J., Williams, T. L., Tennant, A., & group, Ton. study. (2020). Measuring quality of life in ALS/MND: Validation of the WHOQOL-BREF. *Amyotrophic Lateral Sclerosis and Frontotemporal Degeneration*, *21*(5–6), 364–372. https://doi.org/10.1080/21678421.2020.1752244

Zaki, M. A. (2009). Reliability and Validity of the Social Provision Scale (SPS) in the Students of Isfahan University. *Iranian Journal of Psychiatry and Clinical Psychology*, *14*(4), 439–444.
